# Diagnosis of the Dyspepsias
1A Paper read at a Meeting of the Bristol Medico-Chirurgical Society on Wednesday, April 8th, 1925.


**Published:** 1925

**Authors:** A. Rendle Short

**Affiliations:** Surgeon to the Bristol Royal Infirmary


### DIAGNOSIS OF THE DYSPEPSIAS.[1]

BY

A. Rendle Short, M.D., B.S., B.Sc., F.R.C.S.,

*Surgeon to the Bristol Royal Infirmary.*

---

The basis for the present discourse is a recent series of 100 cases of dyspepsia (not including any due to carcinoma) in which the notes were taken to a plan, nearly all examined by the X-ray screen in my presence, all verified by operation, and in nearly all the after-history is known.

Reviewing one's intellectual pilgrimage for the past twenty years, it is evident that the factors making for progress have been a ruthless scrapping of the old classification of the dyspepsias and the recognition of a new one; a better knowledge of physiology; disregard of hæmatemesis in the diagnosis of ulceration; and the regular use of X-ray screening (not plates).

### ADVANCES IN PHYSIOLOGY.

It is now established that the normal stomach may show considerable deviations from the average, quite apart from the presence of any symptoms whatever. There is a hypertonic type, seen in 17 per cent. males and 7 per cent. females, in which the acidity of the gastric juice is high (0.2 per cent.), and the stomach examined by the X-ray screen is small, well braced-up, and empties rather rapidly, say in two hours or less. And there is a hypotonic type, seen in 4 per cent. males and 15 per cent. females, with low gastric acidity (0.1 per cent.), large dropped stomach, and slow emptying, say four or five hours. In about 4 per cent. cases HCl is absent altogether from the gastric juice, apparently without harm (though it is suggested that such

[1] A Paper read at a Meeting of the Bristol Medico-Chirurgical Society on Wednesday, April 8th, 1925.

persons may eventually develop pernicious anæmia). It is often impossible to tell from symptoms whether the patient belongs to the one group or the other. But although these two types are quite consistent with perfect health, there is definite morbidity attached to each. Pushed beyond a certain limit, or tampered with by faulty food habits on the part of the patient, or on account of a failure of the general health, characteristic symptoms appear. Those with the hypertonic stomach suffer from hunger; the hunger-sensations may become actually painful, and a duodenal ulcer, acute or chronic, may develop.

In the hypotonic type the tendency is to develop visceroptosis, constipation, etc.

Modern physiology has further taught us that the alimentary canal is arranged on what might be described as a lock-gate system, the sphincters corresponding to the locks. Traffic is not passed on from above until the all-clear signal is received from below, and *vice versa* when food is taken an effort is made to expedite the traffic farther down the canal. I have seen in a patient with extroversion of the ileo-cæcal valve, which is really a sphincter, a regular gush of ileal contents whenever food was taken by the mouth. This helps us to understand that if on account of appendicular trouble there is delay at the ileo-cæcal sphincter it may reflexly cause delay in the stomach. This has been verified by the delay in emptying of the stomach, by spasm seen with the X-rays, and by writhing visible on the operation table, principally at the pyloric end. In 55 per cent. cases there is also a secretion-reflex in the shape of hyperchlorhydria.

Another point of importance is the demonstration that after the age of four lymphoid nodules can be found in the gastric mucosa, especially along the lesser curvature. They tend to atrophy after middle life. It is these nodules which are apt to ulcerate and bleed.

### HÆMATEMESIS.

It cannot be too strongly emphasised that even a large vomiting of blood is no proof whatever of the presence of a gastric or duodenal ulcer that can be found by operation. Cases of hæmatemesis of gastro-duodenal origin, and excluding splenic anæmia, cirrhosis of the liver, and acute infections, fall into three groups.

1. *Bleeding without gastric symptoms.*—These are mostly in young women, and even at autopsy, if they die (which they seldom do), it is usually difficult to find any source for the hemorrhage. Occasionally one sees a soft shallow ulcer as large as a sixpence, with an eroded artery. Here belong the cases called " gastrostaxis " by some.

2. *Bleeding with gastric symptoms,* such as pain, flatulence, vomiting, etc., but in which operation reveals no ulcer. In my experience of this group one generally finds an atrophic appendix without a lumen.

3. *Bleeding with gastric symptoms, due to gastric or duodenal ulcer,* which can be demonstrated at operation or autopsy.

It may be very difficult to differentiate the second and third of these groups, as the occurrence of the hæmatemesis is common to both. In my series 1 in 3 of the cases of peptic ulcer bled, and 1 in 6 of the cases of appendicular dyspepsia.

It is quite obvious where the blood comes from in the third group, but even at autopsy it may be difficult or impossible to discover the source in the first and second groups. At the London Hospital for three years a special search was made at all post-mortems for *little scars* in the gastric mucosa, and they found 149 small acute ulcers and 59 tiny scars in 3,761 autopsies, *i.e.* 5.5 per cent. The incidence was highest amongst women between eighteen and thirty.

Although these hemorrhages are so often associated with an atrophic appendix which has been undergoing quiet fibrosis, it must not be concluded that appendicectomy will necessarily prevent a return of the bleeding. In one of my cases such a return did occur some years after the operation. Probably the abnormality of the appendix is associated with, rather than causative of, changes in the lymphoid nodules in the gastric mucosa.

### CLASSIFICATION.

The most useful classification of the dyspepsias is into three main groups, with several subdivisions in each. These are :—

1. *Functional Dyspepsia.*—The title is perhaps not quite explicit, but serves to indicate that no gross abnormality will be found either in the stomach or the alimentary canal.

Here belong acute and chronic gastritis from faulty food habits or alcoholism, or defective teeth. Here also the secondary dyspepsias accompanying phthisis, Bright's disease, the grave anæmias, and other serious constitutional disturbances. Here also the frankly hysterical manifestations, such as anorexia nervosa, functional vomiting, and ærophagia. And here yet again patients with hypertonic or hypotonic stomachs, suffering from an exaggeration of their diathesis.

The diagnosis ought not to give serious trouble. *Gastritis* is often diagnosed, seldom seen. It is characterised by much mucus in the vomit. Accessory signs are a furred tongue and deficient HCl in the test meal.

The frequent vomiting of early *phthisis* has to be borne in mind, and can usually be recognised by the signs in the chest.

When a patient, not obviously ill or suffering from intestinal obstruction or other grave disorder, keeps on vomiting for weeks immediately any food or drink is taken but does not seem to lose flesh and there is little pain, it can only be hysterical. These patients are apt to go to every doctor in the town, and to exhaust their welcome everywhere, unless cured by psychotherapy. Aerophagia is another curious neurosis ; the amount of gas which can be evolved in the stomach by fermentation is quite limited, and if a patient goes on belching wind gulp after gulp it is certain that they are merely swallowing air and bringing it up again. Some patients are capable of performing prodigies in this direction, especially if frightened or upset.

Patients with hypertonic stomachs, if they are run down in health or irregular in their meals or smoke too much, will often develop hunger-pain and water-brash. This is too transient and too easily cured by simple dieting and treatment to be attributed to a chronic callous duodenal ulcer ; there may or may not be a tiny soft erosion of the mucous membrane.

The hypotonic group also may suffer from the faults of their diathesis, and when tired or "nervy," or after influenza, they get flatulence, discomfort after meals and a sense of weight and fulness, with constipation. Given a "rest cure," tonics, and support for their dropped viscera, with liquid paraffin for the bowels, they improve, at any rate for a time. Here belong the "atonic dilatation of the stomach" cases of the older books. Both the hypertonic and the hypotonic types are readily recognised by barium skiagraphy. The stomach may be found just as large and dropped in some patients who do not have symptoms as in some who do. There is no difficulty in distinguishing the hypotonic stomach from the dilatation due to pyloric obstruction. The former does not cause vomiting or wasting ; the latter does both. A barium meal resolves all doubts. If the peristalsis is vigorous but nothing gets out through the pylorus, and even after twelve or twenty-four hours most of the meal is still retained, there is definite obstruction. In the hypotonic stomach peristalsis is quiet, but some gets out, and most of the meal will have escaped in twelve hours.

Generally speaking, then, the functional dyspepsias are easily diagnosed, and easily relieved by medical treatment.

2. *Reflex Dyspepsia.*—This is an important and numerous group, much more numerous than the organic dyspepsias due to demonstrable lesions of the stomach or duodenum. Also, it is often difficult to diagnose, and refractory to medical treatment. Therefore the majority of the cases that are referred by the general practitioner to a surgeon belong to this group.

The source of the reflex may be located in the appendix, or in the gall-bladder, or there may be visceroptosis and intestinal stasis with a large, dropped, atonic cæcum, or the cause may be obscure.

It is very characteristic of the reflex dyspepsias that though they give a colourable imitation of the book symptoms of gastric or duodenal ulcer, there is generally some deviation from type. There may be complaint of pain after food, vomiting and hæmatemesis; but a careful history shows, in some of them, that the pain is *continuous*, always present but made rather worse by meals—this is highly suggestive (unless the patient is very ill from advanced cancer or deep ulceration). Or the vomit *may not relieve the pain*, which it almost invariably does in organic gastric disease (except, again, in advanced cancer). Or what is called pain does not really amount to more than discomfort; true ulcer nearly always causes indubitable pain. Hunger-discomfort is often due to reflex dyspepsia; hunger-pain suggests duodenal ulcer. Nevertheless, it must be admitted that sometimes there is real pain, and vomiting may give relief, and the pain may *not* be continuous, but quite definitely related to food.

Test meals do not help us much here, but barium skiagraphy is very valuable. The special signs of ulcer or cancer are not found. There may be some delay in emptying the stomach, but very little will be left after twelve hours. I have sometimes been able to demonstrate that after appendicectomy in such a case the emptying time has returned to normal. There may be reflex spasm nipping the stomach into two pouches, but it does not persist and can be abolished by atropine.

It is more difficult to say which viscus is the source of the reflex. If the *gall-bladder* is at fault the patient is usually a woman over forty, and fat. Flatulence is the main complaint. Discomfort may follow certain special articles of diet, such as cooked fats, or apples—the "qualitative dyspepsia" of American writers. Alkalies do not give relief, as they do in most other stomach disorders. The barium meal may show adhesions of the duodenum to the gall-bladder. Rarely one can see stone-shadows. Of course, if the patient gets an attack of gall-stone colic the nature of the case becomes perfectly clear.

In the appendicular cases there may or may not be tenderness or pain in the right iliac fossa. If there is not, and there is no history of a frank attack of acute appendicitis, the diagnosis is likely to be doubtful. The age and sex are much as in gastric ulcer. They sometimes get two or three attacks of really severe epigastric pain lasting a day or two, without a rise of temperature or any tokens of the appendicular origin. Barium skiagraphy may help by showing great delay at the ileo-cæcal valve, and if the appendix fills with bismuth, it may be tender, or as in one case under my care, verified by operation, there may be a concretion shown by the X-rays.

3. *Organic Dyspepsias.*—Here belong gastric ulcer, duodenal ulcer, cancer of the stomach, pyloric stenosis, peri-duodenal adhesions, duodenal ileus, and leather-bottle stomach. Hour-glass stomach is merely a special case of gastric ulcer. Duodenal carcinoma does occur, but is rare and probably not diagnosable.

The organic dyspepsias, in comparison with those we have already discussed, are not very common, except, unfortunately, cancer.

*Gastric ulcer.*—The special feature in the diagnosis of this condition is the regular course of the symptoms, which may be conveniently remembered as the F.R.P.V.R. sequence. That is to say, they take *food*, then there is a period of *relief*, which in about a fourth of my cases lasted under half an hour but in three-fourths lasted from half an hour to an hour or more. Then comes pain, which may eventually be followed by *vomiting*, and the vomiting gives marked *relief* again. This regular sequence is of outstanding importance in arriving at a diagnosis. Let it be observed that they get *pain*, and not merely discomfort. Usually it is epigastric, but in five of my patients it was in the lower abdomen, and once or twice the diagnosis made was cancer of the colon. Often the history of pain is of many years' standing. Late in the case the pain changes in character and becomes continuous, and bores through into the back. This nearly always means, in my experience, that there is a deep ulcer invading the pancreas.

Patients with gastric ulcer nearly all vomit, but the frequency of the vomiting varies exceedingly. It does not usually come on until an hour or more after a meal. Hæmatemesis occurs in about a third of the cases, but it may have been many years ago. Occult blood can generally be found by repeated examination, either in the stools or fæces, but its diagnostic value is not very great.

In my experience the test meal is of little value in the recognition of gastric ulcer. The HCl may be excessive, normal, or subnormal with equal frequency.

When symptoms of ulcer of the stomach have been present for seven years or more, and there is considerable emaciation, one may wisely suspect hour-glass stomach, but it cannot usually be recognised with confidence apart from skiagraphy.

The barium meal and X-ray screening often give great help in the diagnosis, but considerable clinical experience and much patience are required to avoid bad mistakes. Small soft ulcers are usually missed. Carman states that they can be found by watching the stream of barium entering the stomach, but I have not met with success yet by this method. Ulcers causing stenosis of the pylorus give rise to the evidences of dilated stomach with pyloric obstruction, which have already been discussed, but it will probably be impossible on X-ray signs alone to distinguish between ulcer, cancer, and simple stenosis at the pylorus. Gastric ulcers not at the pylorus show three principal signs: six-hour retention of the bulk of the meal; an islet or niche, where the barium has lodged in the cavity of a fairly large ulcer, and spasm pinching the stomach nearly across, from the greater curvature towards the lesser, just proximal to the ulcer. It is necessary to examine several times, at various periods after the meal, and to turn the stomach about with the hands, to demonstrate these signs. Several times I have been put on the right track by finding all the meal in the proximal pouch, giving an appearance of the stomach being too far to the left. Six-hour retention, by itself, is not diagnostic, and one needs to distinguish between the transient spasm occasionally seen in reflex dyspepsia and the intractable spasm of gastric ulcer. The X-ray picture of hour-glass stomach is perfectly distinctive, though the tyro may be deceived by the loculation of the shadow due to pressure of the bodies of the vertebræ on the normal stomach. I have seen ludicrous mistakes made in this way.

*Duodenal ulcer.*—As is well known, this disease is much commoner in males than females (four to one in my series). The mnemonic is F.R.P. That is to say, the characteristic sequence is *food*, followed by a long *relief*, generally one and a half to two hours or more, then *pain*, which is severe, and so can usually be distinguished from the hunger discomfort of reflex dyspepsia or the merely hypertonic stomach. The relief after food is more definite and longer than in the case of gastric ulcer. They may get fat from over-eating in consequence. Vomiting is generally at long intervals only, and half of my cases never vomited at all. In old-standing cases, where the duodenum is narrowing, it is more in evidence. Water-brash is rather common. About a third of the patients get hæmatemesis.

A curious and unexplained feature both of gastric and duodenal ulcer is *periodicity*, that is to say they have periods of several weeks or months with symptoms, and then longer or shorter intervals free from all trouble. This does not mean that the ulcer has healed. It is of no great diagnostic value; such periodicity is occasionally seen in reflex dyspepsia, and is not constant with ulcer, though common.

The test meal nearly always shows hyperchlorhydria, but as this so often occurs in the absence of ulceration, it is not very helpful.

The X-ray diagnosis of duodenal ulcer is often conclusive enough, but requires unusual care and experience, and even so a negative finding does not exclude it. The decisive tokens of ulcer are persistent deformity of the duodenal cap together with hurried emptying of the stomach. Probably this hurry is merely due to the underlying hypertonic diathesis; the peristalsis is seen to be very active, and the meal may have passed entirely out of the stomach in an hour.

The deformity of the duodenal cap is due to spasm in the first part of the duodenum. Sometimes one can detect it with the screen, but the best method is to catch the duodenal cap full by screening, and then to take an instantaneous photo. It is necessary to take care that the shape of the cap is not merely due to incomplete filling.

In cases in which the cap cannot be got into view or no deformity is present there may be evidence of duodenal abnormality, in that the barium instead of taking the second and third parts of the duodenum at a rush, which is normal, passes through this region of the bowel with extreme slowness, and may dance up and down. This sign does not prove duodenal ulcer, but in my experience it is definitely pathological, and in the presence of marked symptoms warrants exploration. One may find duodenal ulcer, duodenal ileus, gastric ulcer, or adhesions to the gall-bladder.

*Duodenal ileus.*—This condition was elevated to the dignity of description as a pathological entity by Wilkie two or three years ago, and I think rightly. It is common and important. The second and third parts of the duodenum are greatly distended and may be almost as big as the stomach; the distension ends rather suddenly where the superior mesenteric vessels cross the duodenum. The symptoms are sufficiently definite to enable one to suspect its presence on the history, and it can be diagnosed conclusively by X-rays. My students have several times made the diagnosis correctly. The nature of the condition requires further study. It certainly seems that in some cases, at any rate, the pressure of the vessels does produce a real obstruction, and duodeno-jejunostomy is curative. I have had some very successful examples of this, followed up for a year or so, but the treatment is new, even in Wilkie's hands, and time must test its value. Sometimes the dilatation of the duodenum is part and parcel of a general visceroptosis and intestinal stasis, though even here the drag of an over-distended and dropped cæcum will stretch the superior mesenteric artery across the duodenum. It may be, however, that duodenal ileus is merely a semi-paralytic condition due to loss of tonus.

The symptoms vary somewhat, but most commonly there is flatulence and nausea, both very persistent even for years, accompanied often but not always by vomiting and discomfort, which seldom amounts to pain. The relationship to food is apt to be vague. Thus it mimics the reflex dyspepsias. X-ray screening shows great delay in the passage of the meal through the second and third parts of the duodenum, associated with immense distension of the loop formed by them. Much of the meal may be hung up here even after the stomach and small intestines are empty.

*Peri-duodenal adhesions.*—This condition is found so frequently as the sole explanation of an inveterate dyspepsia that it ought for the sake of accuracy to be reckoned as a possible diagnosis. That the diagnosis is possible is shown by the fact that in my last case it had been correctly made by a physician, a radiographer, and a surgeon all working independently. The outstanding features were a long history, much pain late after a meal, vomiting followed by relief, freedom from pain when the stomach was empty, no hæmatemesis, and a big splashy stomach easily felt. The X-ray showed great delay in emptying (twelve to twenty-four hours) and persistent spasm of the duodenum. In some of my cases even a copious drink of water gave rise to the pain. The skiagram may show that the pylorus is pulled far over to the right, and there may be stasis in the second and third parts of the duodenum. Division of the adhesions cures the condition, thereby proving that it is a genuine cause of trouble.

*Cancer of the stomach.*—For the sake of completeness this is mentioned, but carcinoma was excluded from the series forming the basis of this study, and the topic is too complicated to discuss in detail, important as it is. We will merely point out that cancer of the stomach is common in men after forty-five, and ought always to be borne in mind when a man over that age develops an intractable dyspepsia.

The well-read practitioner will notice my indebtedness to the recent teachings of Sir Berkeley Moynihan, Carman and other members of the Mayo clinic, Hurst, Walton and Wilkie.

DISCUSSION.

The PRESIDENT appeared to think that a better title would have been "Diagnosis of *Surgical* Dyspepsia"; only a small proportion of dyspepsia was amenable to surgery. A great proportion was due to worry. He viewed with distrust cases of dyspepsia due to kinks, veils and malpositions of viscera, but he agreed with Mr. Short as to the uncertain value of a test meal in gastric cancer and the importance to be attached to anorexia.

MR. C. A. MOORE asked what were the symptoms and pathology of duodenal ileus. He thought deformity of the duodenal "cap" rare. He related a case illustrative of the great difficulty of diagnosing gall-stone from duodenal ulcer. He agreed with Mr. Short that an atrophic appendix may be the cause of dyspepsia, and also that the dyspepsia may be accompanied by hæmatemesis.

DR. D. A. ALEXANDER spoke of the value of long-kept records in diagnosis of dyspepsia, and instanced cases of tuberculous adhesions in point.

MR. A. W. ADAMS agreed with Mr. Short's observation as to duodenal ileus. He found this condition in one case diagnosed chronic appendicitis. He suggested that this condition might be associated with those cases of acute post-operative gastric dilatation, the mesenteric vessels by compressing the duodenum being responsible.

Mr. Short, in reply, said that though duodenal ileus was difficult to diagnose before operation, in a typical case this was possible. He was glad others agreed that dyspepsia and hæmatemesis occurred as symptoms of appendicitis.

---

